# A neural circuit encoding mating states tunes defensive behavior in *Drosophila*

**DOI:** 10.1038/s41467-020-17771-8

**Published:** 2020-08-07

**Authors:** Chenxi Liu, Bei Zhang, Liwei Zhang, Tingting Yang, Zhewei Zhang, Zihua Gao, Wei Zhang

**Affiliations:** School of Life Sciences, Tsinghua-Peking Joint Center for Life Sciences, IDG/McGovern Institute for Brain Research, Tsinghua University, 100084 Beijing, China

**Keywords:** Neural circuits, Sexual behaviour

## Abstract

Social context can dampen or amplify the perception of touch, and touch in turn conveys nuanced social information. However, the neural mechanism behind social regulation of mechanosensation is largely elusive. Here we report that fruit flies exhibit a strong defensive response to mechanical stimuli to their wings. In contrast, virgin female flies being courted by a male show a compromised defensive response to the stimuli, but following mating the response is enhanced. This state-dependent switch is mediated by a functional reconfiguration of a neural circuit labelled with the *Tmc-L* gene in the ventral nerve cord. The circuit receives excitatory inputs from peripheral mechanoreceptors and coordinates the defensive response. While male cues suppress it via a *doublesex* (*dsx*) neuronal pathway, mating sensitizes it by stimulating a group of uterine neurons and consequently activating a leucokinin-dependent pathway. Such a modulation is crucial for the balance between defense against body contacts and sexual receptivity.

## Abstract (web summary)

Wing touching induces a defensive response in *D. melanogaster*. Here, the authors show that female flies change the defensive response during courtship and after mating. This switch is mediated by functional reconfiguration of a neural circuit in the ventral nerve cord.

## Introduction

Touch can be pleasant or aversive. Animals’ responses to external stimuli, including mechanical force, are tuned by internal states and social contexts^1,2^. The perception of touch is affected with social context and in turn conveys nuanced social information. This versatility of mechanosensory behaviors is essential for animals to adapt to different environments. These modulations can occur at both the peripheral and central nervous systems^3,4^. However, the neural mechanism behind social regulation of mechanosensation is largely elusive.

In animals with intra-species interaction, response to body touch is actively regulated with social contexts^5^. For example, activation of mechanosensory neurons on the fore-leg induced by tapping can trigger collective behavior in *Drosophila*^6^. During courtship, a major form of social interaction between flies, the role of touch sensation is poorly understood. Unlike visual, auditory and chemical cues known to play important roles during sexual behaviors of flies, the tactile communication between a pair of flies during courtship is often overlooked. One reason is that body touch can occur to most parts of the body surface and the females’ response is highly diverse^7–9^, and unlike other sensory modes, there are multiple regions in the brain and the ventral nerve cord that receive mechanosensory inputs^10,11^. Moreover, mechanical stimuli on the body surface are usually alert signals and flies tend to provoke escape or defensive response^12^. This gives rise to a profound question: how do female flies adjust their responsive state to body touch according to the context of sexual activity? In female mice, different groups of neurons in the ventromedial hypothalamus seem to play different roles in regulating sexual receptivity and defensive behaviors^13^, implicating that defensive response is affected with mating activities. However, the dissection of neural circuits that mediate the interplay between mating states and sensory inputs has not been achieved.

In recent years, progress has been made to uncover the genetic and neuronal basis of sexual behaviors, although the studies on males far outnumber those on females. Female flies have well-established sexually dimorphic behaviors that are under rigorous control. For instance, the *doublesex* (*dsx*) circuits in the brain are activated with male pheromone and courtship song to slow down the female’s locomotion^14,15^. Meanwhile, an increase of receptivity causes a reduced level of kicking and fencing against male’s tap or lick^8^. In contrast, mating can trigger a switch of both behaviors and internal states in female, including reduced receptivity and changes in diet preference^16,17^. They also become more aggressive when competing for food resources^18^. Whether mated female flies become more responsive to or alert against body contact stimuli compared to pre-mated females is unknown.

To answer these questions, we combine genetic and behavioral approaches to investigate the versatility of female flies’ defensive response to body contacts. We identify a somatosensory center in the ventral nerve cord that transmits somatosensory input to motor actions. We find that the functional plasticity of this neural structure is responsible for the mating-induced switch in defensive response. We reveal the neural basis for the sexual activity inducing modulation of touch sensitivity.

## Results

### A VNC circuit mediates defensive response in female flies

**Fig. 1:**
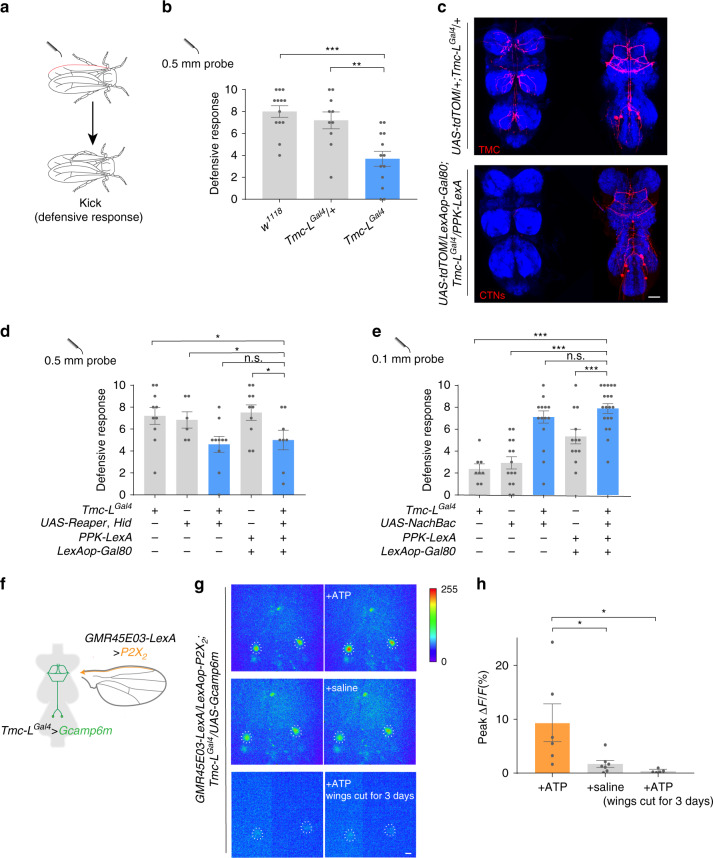
Central *Tmc-L* neurons (CTNs) mediate defensive response to mechanical stimuli on the wing margin. **a** Schematic of the defensive response assay. A probe was used to challenge the mechanosensory bristles on the wing margin (marked with red) of decapitated flies. **b** Defensive response score of *w*^*1118*^, *Tmc-L*^*Gal4*^*/+*, and *Tmc-L*^*Gal4*^ stimulated 10 times by a 0.5 mm probe. *n* = 13, 10, and 13 for each group. **p < 0.01 and ***p < 0.001, two-tailed unpaired *t* tests. **c** Expression pattern of *Tmc-L*^*Gal4*^ (red) in the ventral and dorsal sides of VNC. Lower panel: *PPK-LexA* > *lexAop-Gal80* inhibited peripheral projections of *Tmc-L* neurons to the ventral VNC. Blue: nc82. Scale bar represented 50 μm. **d** Ablating all *Tmc-L* neurons or only CTNs with Reaper and Hid. *n* = 10, 6, 10, 10, and 8 for each group. *p < 0.05, *p* > 0.05 (n.s.), two-tailed unpaired *t* tests. **e** Activating all *Tmc-L* neuron or only CTNs with NachBac. *n* = 8, 14, 18, 12, and 19 for each group. ***p < 0.001, *p* > 0.05 (n.s.), two-tailed unpaired *t* tests. **f** Schematic of the Ca^2+^ imaging assay of (**g**). **g** Calcium response of CTNs’ cell bodies in the VNC before (left) and after (right) application of ATP or saline. Cell bodies of CTNs were cycled with white dashed circles. Scale bar represented 50 μm. **h** Peak fluorescence changes (Δ*F*/*F*) of CTNs’ cell bodies after ATP application (*n* = 6), saline application (*n* = 7) and ATP application to VNC from flies whose wings had ablated for 3 days (*n* = 5). *p < 0.05, two-tailed unpaired *t* tests. Error bars indicate mean ± SEM, n.s., not significant. Source data are provided as a Source Data file.

Flies exhibit a robust defensive response to physical contacts on their bodies that typically alerts them to harmful contaminations or parasitic invasions^12^. These are detected by specialized mechanosensors distributing over the body surface and usually trigger self-cleaning behaviors like grooming or defensive response such as a swift kick^12^. To quantify the defensive response, we touched the mechanosensory bristles on the wing margin of decapitated flies with a fine probe and counted the kicking number out of ten touches (Fig. 1a). As previously reported^12^, wild-type flies exhibited stereotyped kicking behavior against the touch on their wing margins, thus we defined this kicking behavior as the defensive response. Decapitated flies kicked as precisely and quickly as the intact flies did, so we used decapitated flies to test the defensive response in most experiments (see below). The propensity of the defensive response increased with the diameter of the stimulating probe (Supplementary Fig. 1a).

Strikingly, we found that a mutant for the long isoform of the *transmembrane channel-like* (*Tmc-L*) gene showed a much-reduced defensive response, indicating that the *Tmc-L* gene was involved in this behavior (Fig. 1b). To delineate the functions of the *Tmc-L* neurons, we first ablated them with Reaper and Hid, two apoptosis triggering proteins^19^. The defensive response was noticeably lower than control groups (Fig. 1d), confirming that the *Tmc-L* neurons was important for the defensive response.

In the periphery, *Tmc-L*^*Gal4*^, a knock-in driver line for *Tmc-L*, marked sensory structures on the appendages (Supplementary Fig. 1b). In the ventral nerve cord (VNC), this Gal4 line labeled signals of both peripheral projections and central neuronal cell bodies (Fig. 1c). To pinpoint the component in the *Tmc-L* circuits that mediated the defensive response, we used a *PPK-lexA* > *lexAop-Gal80* line to suppress the peripheral expression of the *Tmc-L*^*Gal4*^ (Supplementary Fig. 1b). This refinement revealed that the *Tmc-L*^*Gal4*^ labeled 4–6 neurons (henceforth termed as central *Tmc-L* neurons (CTNs)) in the metathoracic ganglion of the VNC, and these neurons had ascending neurites projecting to the accessory mesothoracic ganglion. The overall morphology of CTNs resembles a music stand shape (Fig. 1c).

With the same intersection strategy, we expressed Reaper and Hid to ablate CTNs. As a result, the defensive response against touch on the wing margin was significantly reduced compared with controls, indicating that this small subset of *Tmc-L* neurons in the VNC were involved in the defensive response (Fig. 1d). Conversely, when we elevated these neurons’ activity by Gal4 driven overexpression of NachBac, a bacterial voltage-gated sodium channel^20^, the flies exhibited increased defensive response to the same mechanical stimuli delivered with a 0.1 mm probe that normally triggered a low level of defensive response (Fig. 1e and Supplementary Fig. 1a). These results demonstrated that CTNs were a part of the somatosensory circuit mediating the response to mechanical stimuli on the wings.

### CTNs are activated by mechanoreceptors along the wing margins

From these results, we speculated that CTNs received inputs from mechanoreceptors on the wing margins and then guided the defensive response. We first screened for driver lines that specifically label the mechanoreceptors on the wing margin and found the line *GMR45E03-Gal4*. This line marked putative mechanosensitive neurons associated with each recurved bristle along the wing margin that send their axons to the *Nanchung* neuropil in the accessory mesothoracic ganglion of the VNC^12^ (Supplementary Fig. 1c). Blocking these neurons’ activity largely reduced the defensive response against wing margin touch (Supplementary Fig. 1d). To explore the functional connectivity between wing margin mechanoreceptors and CTNs, we used the ATP/P2X_2_ system^21^ in an ex vivo preparation to stimulate the wing margin mechanosensory neurons and simultaneously recorded Ca^2+^ influx in CTNs using GCaMP6m^22^ (Fig. 1f). Upon application of ATP, strong Ca^2+^ responses were elicited in CTNs (Fig. 1g). The response was absent in saline only controls or in the flies whose wings were cut three day prior to the imaging experiments (Fig. 1g, h). The same stimulation did not elicit detectable Ca^2+^ responses in the genetic control animals (Supplementary Fig. 1e). The fluorescence of heterozygous Tmc-L driver expressed GCamp6m was too weak to resolve all the 4–6 neurons, thus we quantified the two superficial neurons for consistence. These results support the notion that CTNs integrate mechanosensory input from the periphery for the control of defensive actions, although an explant may not completely reflect the response to wing touch in intact flies.

### Defensive response is suppressed during courtship

**Fig. 2:**
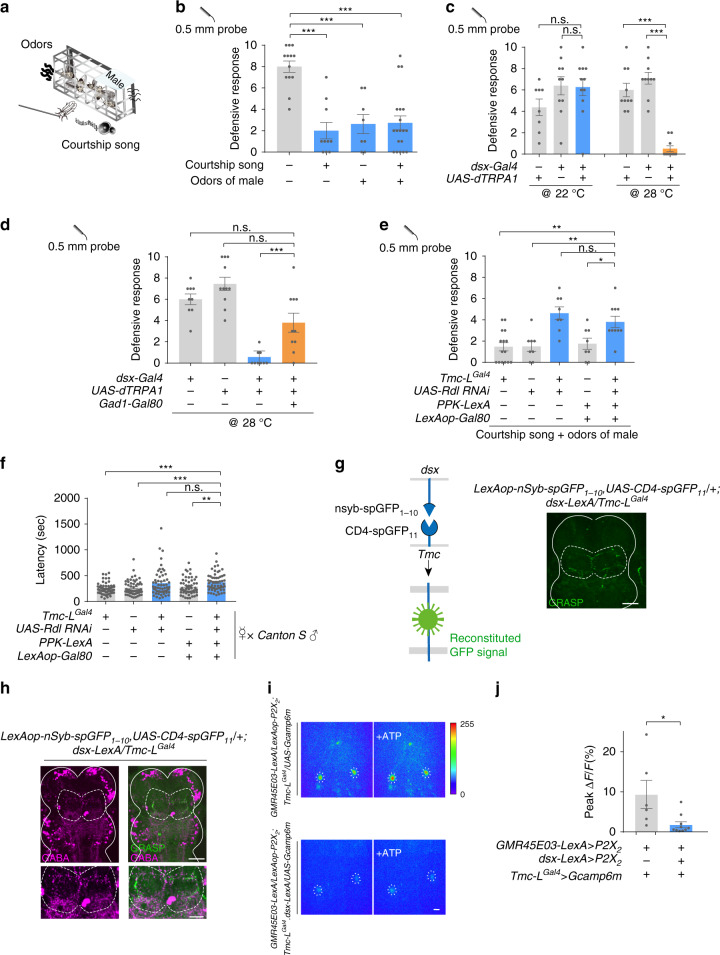
Male cues suppress defensive response by activating *dsx* neurons. **a** Schematic of simulative courtship assay. Tethered virgin flies were placed on top of a speaker playing back courtship song and 1 cm away from the chamber with males. Virgin flies were challenged with mechanical stimuli on their wing margin. **b** Wild-type (*w*^*1118*^) virgins placed either in the proximity of a group of male flies or a speaker playing fly courtship song had decreased defensive response. *n* = 13, 10, 8, and 19 for each group. ***p < 0.001, two-tailed unpaired *t* tests. **c** Activating *dsx* neuron with dTrpA1 in decapitated virgins caused decreased defensive response against mechanical stimuli. *n* = 9, 10, 11, 10, 10, and 10 (from left to right). ***p < 0.001, *p* > 0.05 (n.s.), two-tailed unpaired *t* tests. **d** Activating *dsx* neurons that do not release GABA in decapitated flies did not suppress defensive response. *n* = 9, 11, 9, and 10 for each group. ***p < 0.001, *p* > 0.05 (n.s.), two-tailed unpaired *t* tests. **e** Knocking-down GABA_A_ receptor in all *Tmc-L* neurons or only CTNs with *Rdl-RNAi* did not suppress defensive response to mechanical stimuli in the virgin female flies exposed to courtship cues. *n* = 15, 8, 8, 8, and 10 for each group. **p < 0. 01, *p < 0. 05, *p* > 0.05 (n.s.), two-tailed unpaired *t* tests. **f** Knocking-down GABA receptor in all *Tmc-L* neurons or only CTNs with *Rdl-RNAi* in virgins prolonged time to copulation, and these virgins were paired with naïve *Canton S* male. *n* = 58, 55, 58, 57, 55 for each group. ***p < 0.001, **p < 0. 01, *p* > 0.05 (n.s.), two-tailed Mann–Whitney nonparametric test. **g** GRASP signal (green) between *dsx* neurons and CTNs in female VNC highlighted with white dashed line. White line outlined the border of the mesothoracic ganglion of the VNC. Scale bar represented 50 μm. **h** GRASP signal (green) between *dsx* neurons and CTNs in female VNC was positive to GABA immune-staining (magenta). GRASP signal was highlighted with white dashed line. GRASP signal region was zoomed in below. Scale bar represented 50 μm (upper) and 25 μm (lower). **i** Activation of wing mechanosensory neurons via P2X_2_ triggered a robust Ca^2+^ influx in CTNs’ cell bodies (upper). The influx was inhibited when wing margin mechanosensory neurons and *dsx* neurons were activated simultaneously (lower). The cell bodies of CTNs were cycled with white dashed lines. Scale bar represented 50 μm. **j** Peak fluorescence changes (Δ*F*/*F*) of CTNs’ cell bodies when activating wing margin mechanosensory neurons (left bar, *n* = 6), or activating wing margin mechanosensory neurons and *dsx* neurons simultaneously (right bar, *n* = 10). *p < 0. 05, two-tailed Mann–Whitney nonparametric test. Experiments in **d**–**f**, **i**, **j**) were done with virgins. Error bars indicate mean ± SEM, n.s., not significant. Source data are provided as a Source Data file.

Defensive response against harmful body contacts is a critical survival instinct for flies. However, flies may need to suppress this response temporarily under certain circumstances, for instance, during courtship when they receive intense body contacts from their suitors^23^. The chance of successful mating would be minimal if females showed persistent defensive actions to body contacts from the courting male. Thus, we speculated that the defensive response of females may be suppressed during courtship. To test this, we studied tethered virgin flies which were challenged with mechanical stimuli on their wing margin and scored the defensive response. The tethered female flies showed robust defensive response under this preparation (Fig. 2b). However, when the virgin flies were placed in the proximity of a group of male flies or a speaker playing a male courtship song (Fig. 2a), their defensive response to the same stimuli was markedly reduced (Fig. 2b), indicating that male cues such as odor or song produced during courtship suppressed the defensive response. We tested whether cVA, a male-specific volatile pheromone^24^, worked as male-associated odor cue that inhibits defensive response in females. Virgins painted with cVA did not show decreased defensive response compared with control groups, indicating that cVA was dispensable for this regulation (Supplementary Fig. 1f). Moreover, this inhibition was absent in males as placing moving virgin females around them did not affect the defensive response (Supplementary Fig. 2d), although the expression pattern of *Tmc-L* (Supplementary Fig. 2a) and function of CTNs in mediating the defensive response in males (Supplementary Fig. 2b, c) appeared similar to those of females.

We thus postulated that this sex-specificity in behavioral suppression may arise from a sex-specific neural inhibition impinging on the defense pathway generated under the control of a neural circuit that regulates female sexual receptivity. *dsx* positive neurons were reported to mediate increased female receptivity during courtship^14^, so we tested whether activation of the *dsx* neurons may suppress the defensive response in decapitated flies. By expressing the warm activated cation channel dTRPA1^25^ under the control of *dsx-Gal4*, *dsx* neurons were activated at 28 °C, resulting in a much lower defensive response. By contrast, the same flies at 22 °C showed a normal defensive response. Gal4 or UAS control flies exhibited a high level of defensive response even at 28 °C similar with those at 22 °C (Fig. 2c). It was reported that activation of *dsx* neurons caused male-like courtship, increase locomotion and elevated receptivity in intact females^14,15,26,27^. Although we did not observe these behaviors in decapitated flies when the *dsx* neurons were activated, it’s possible that the decreased defensive response induced by *dsx* neuron activation in intact flies may partially result from an increase of other behaviors. Notably, activation of *dsx* neurons in male flies did not cause a change of the defensive response, suggesting that this modulation is absent in male flies (Supplementary Fig. 2e).

### *Dsx* neurons inhibit CTNs via GABAergic synapses

*dsx-Gal4* labels a large population of GABAergic neurons in the VNC^28^, so we asked whether suppression of the defensive response by *dsx* neuron activation is mediated by GABA release. When we used a similar experimental setting as above (Fig. 2c) to activate *dsx* neurons but excluding the GABAergic subpopulation with *Gad1-Gal80*, the suppressive effect on the defensive response was abolished (Fig. 2d). We then tested whether CTNs receive direct inhibitory inputs from *dsx* neurons. When the GABA_A_ receptor (*Rdl* in *Drosophila*) was knocked down in CTNs using RNAi, playback of courtship song and the presence of males can no longer suppress the defensive response to mechanical stimuli in the virgin female flies (Fig. 2e). Furthermore, knocking down *Rdl* in CTNs prolonged the time from courtship initiation to copulation (Fig. 2f), suggesting that the suppressed defensive response during courtship facilitated copulation.

Using GFP reconstitution across synaptic patterners (GRASP)^29^, we observed GFP signals along the sheet-holder shaped arborizations of CTNs (Fig. 2g), suggesting that *dsx* neurons and CTNs form synapses in this region. Importantly, the GRASP signal in the female VNC substantially overlapped with GABA immunoreactive materials, suggesting the presence of GABAergic inhibitory synapses (Fig. 2h). This GRASP signal was absent in male flies (Supplementary Fig. 2f), which is in parallel with the behavioral observation that this modulation is absent in male flies (Supplementary Fig. 2e). The above results are consistent with the hypothesis that the courtship cues inhibit CTNs via GABAergic *dsx* neurons in females. We checked the arborization patterns of *dsx* and *Tmc-L* neurons in the metathoracic ganglion of the VNC and found that the projections of *dsx* neurons were dimorphic in that area while *Tmc-L* neurons were not. In females, *dsx* neurons’ neurites were largely overlapped with that of *Tmc-L* neurons in the sheet-holder region. In contrast, the two structures were well separate in males (Supplementary Fig. 2g). These results explained why the GRASP signals between the *dsx* neurons and CTNs only observed in females.

The Ca^2+^ influx in the cell bodies of CTNs triggered with the activation of wing mechanosensory neurons was inhibited when *dsx* neurons were additionally activated at the same time (Fig. 2i, j), demonstrating that *dsx* neurons imposed an inhibitory effect on CTNs to shunt the excitatory inputs induced by male courtship cues. Genetic controls showed no response upon the application of ATP (Supplementary Fig. 1e). Again, the modulation was female-specific, as Ca^2+^ rises in response to stimulation of the wing margin mechanosensory neurons were unaffected by the simultaneous activation of *dsx* neurons in males (Supplementary Fig. 2h). Together, these findings revealed that although CTNs function similarly in both sexes in coordinating the defensive response, there are regulated in a sexually dimorphic manner.

### Mating facilitates the defensive response

**Fig. 3:**
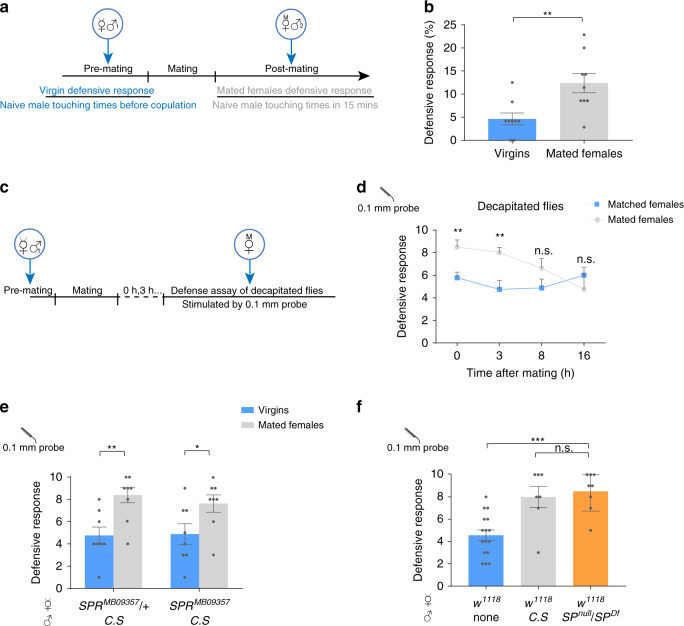
Mating facilitates defensive response independent with SP. **a** Schematic for assays in (**b**). Defensive response ratio of virgin flies and newly mated females were tested for virgin or mated female defensive response stimulated by naïve males divided by naive male touching times. Male touching times to virgins were counted before copulation, and male touching times to mated females were record for 15 min. **b** Defensive response ratio was enhanced in newly mated females. *n* = 9 for each goup. **p < 0. 01, two-tailed unpaired *t* tests. **c** Schematic for assays in **d**. Males were removed after copulation, and mated females were kept for given hours before the defensive response assay. **d** Copulation-induced increase of defensive response lasted for more than 3 h in decapitated flies. Matched females represented that virgins were placed solely in courtship chambers paralleled with mated females under same preparation. *n* = 9, 12, 15, and 8 for each group of matched virgins, *n* = 8, 17, 8, and 8 for each group of mated virgins. **p < 0.01, *p* > 0.05 (n.s.), two-tailed unpaired *t* tests. **e** Female flies of *SPR* mutant alleles, *SPR*^*MB09357*^, showed an increase of defensive response after mating. *n* = 8 for each group. **p < 0. 01, *p < 0.05, two-tailed unpaired *t* tests. **f** Virgins mated with *SP*^*null*^*/SP*^*Df*^ mutant males had a similar post-mating defensive response with virgins mated to *Canton S* males. *n* = 16, 7 and 8 for each group. ***p < 0.001, *p* > 0.05 (n.s.), two-tailed unpaired *t* tests. Error bars indicate mean ± SEM, n.s., not significant. Source data are provided as a Source Data file.

Copulation and male ejaculates induce post-mating response (PMR) in the female flies^30–32^. Reduced receptivity to courtship is among the major behavioral changes of PMR and the suppression emerges in two phases: the fast phase commences immediately after mating, whereas the slow phase takes hours after mating to appear and can last for as long as two weeks^17^. Kicking is one of stereotyped rejection actions displayed by unreceptive females^7^. We thus asked whether the CTNs-mediated defensive response is facilitated by mating by counting the number of female’s kick against male’s body contact during courtship (Fig. 3a). When a sexually mature virgin fly was courted by a male, she rarely kicked against the male’s taps on her wings (Fig. 3b). In contrast, the male contact often elicited the defensive kicking responses from a newly mated female (Fig. 3b). To ask whether the brain is involved in this behavioral change, we repeated the assay for defensive response using decapitated flies at different mating stages (Fig. 3c). Mechanical stimuli induced by the probe on the wing margins caused kicking more often in newly mated decapitated females than the matched group (Fig. 3d), suggesting that the circuit within the VNC is responsible for the change in kicking responses upon mating. The mating-induced increase of the defensive response lasted for hours in decapitated flies (Fig. 3d), similar with the persistence of altered kicking responses in intact flies (Supplementary Fig. 3a, b).

Previous studies identified that sex peptide (SP) in the male seminal fluid that acts on the female SP receptor (SPR) on the sex peptide sensory neurons (SPSN) to regulate PMR^17,33,34^. However, we observed that female flies of several *SPR* mutant alleles still showed an increase in the defensive response after mating (Fig. 3e and Supplementary Fig. 3c, d). Similarly, mating with *SP*^*null/Df*^ mutant males similarly enhanced the post-mating kicking responses in mated females (Fig. 3f), indicating that the SP-SPR pathway is not involved in the observed short-term post-mating modulation of defensive responses.

### *Gr32a* uterine neurons mediate the post-mating switch of defensive responses

**Fig. 4:**
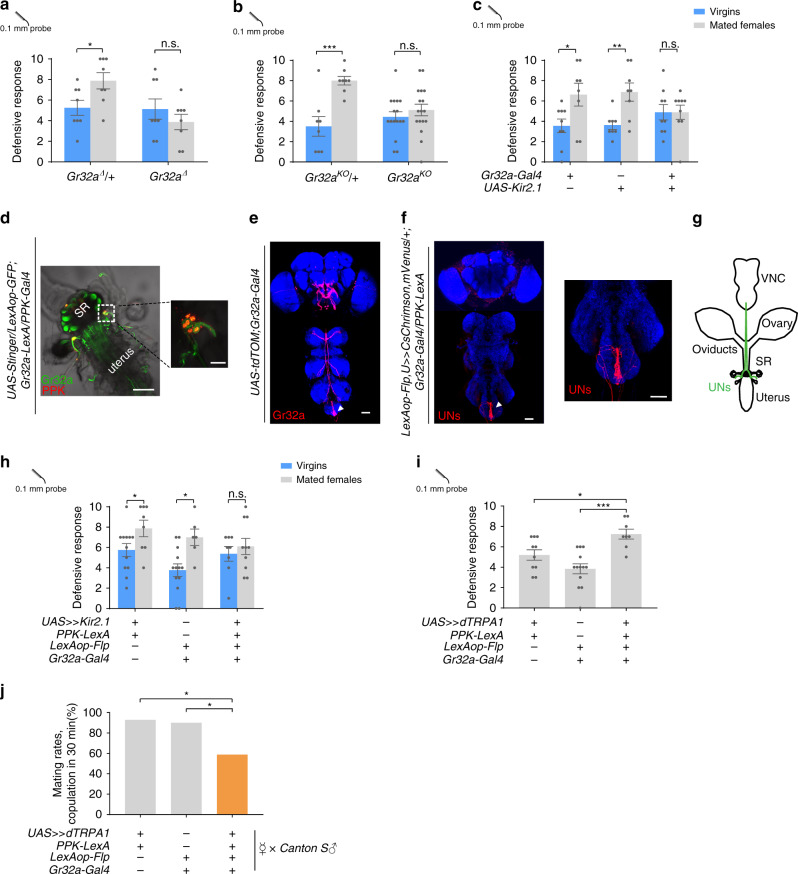
*Gr32a* uterine neurons (UNs) mediate post-mating switch of defensive response. **a**, **b** Two mutant alleles of *Gr32a*, *Gr32a*^*Δ*^(a), and *Gr32a*^*KO*^(b), failed to show the increase defense after mating. *n* = 8 for each group in (**a**), *n* = 8, 8, 16 and 17 (from left to right) in (**b**). ***p < 0.001, *p < 0.05, *p* > 0.05 (n.s.), two-tailed unpaired *t* tests. **c** Silencing *Gr32a* neurons with Kir2.1 eliminated the mating-induced elevation of defensive response. *n* = 9, 8, 8, 8, 9, and 9 (from left to right). **p < 0.01, *p < 0.05, *p* > 0.05 (n.s.), two-tailed unpaired *t* tests. **d** Co-expression of PPK (red) and Gr32a (green) in female reproductive tract. Scale bar represented 50 μm. Right: the cell bodies of PPK (red) and Gr32a (green) on uterus were enlarged. SR indicated seminal receptacle. Scale bar represented 20 μm. **e** Immunofluorescence of Gr32a (red) in the central nervous system. White arrow indicated projections of *Gr32a* neurons on the uterus. Blue: nc82. Scale bar represented 50 μm. **f** Immunofluorescence of UNs (red). The expression of Gr32a in chemoreceptors on other appendages was eliminated by intersection strategy of indicated genotype. White arrow indicated projections of *Gr32a* UNs. Right panel: projections of UNs in the Abg. Blue: nc82. Scale bar represented 50 μm. **g** Schematic showed that the cell bodies of UNs. These UNs projected to the abdominal ganglion of the VNC (green). **h**, **i** Silencing UNs **h** blocked the post-mating enhancement of defensive response, while activating them **i** elevated defensive response in decapitated virgins. *n* = 12, 8, 13, 6, 8, 10 (from left to right) in (h), *n* = 10, 13, 8 for each group in (i). ***p < 0.001, *p < 0.05, *p* > 0.05 (n.s.), two-tailed unpaired *t* tests. **j** Activation of UNs in virgin females decreased the courtship successful rates, and these virgins were paired with naïve *Canton S* male. *n* = 14, 20 and 17 for each group. *p < 0.03, two-tailed Fisher’s exact test. Error bars indicate mean ± SEM, n.s., not significant. Source data are provided as a Source Data file.

What is the mechanism whereby the defensive response increases after mating? We examined the post-mating change in defensive response of females that are mutant for several pheromone receptor genes, based on the assumption that chemical communication between the female and male flies might play a role in this process. We found that gustatory receptor 32a (*Gr32a*) mutant females failed to increase the defensive response after copulation (Fig. 4a, b). Similarly, silencing *Gr32a* neurons with Kir2.1 hampered the mating-induced elevation of the defensive response (Fig. 4c). *Gr32a* is expressed in chemoreceptors on multiple appendages of the fly^35^ (Fig. 4e). Interestingly, a cluster of *Gr32a* neurons is also located on the uterus of the female flies^36^ (Fig. 4d). We thus speculated that these neurons were being activated by copulation and were responsible for increased defensive response. We noticed that the *Gr32a* uterine neurons (termed UNs) were also labeled by *PPK-Gal4* (Fig. 4d), suggesting that UNs are both *Gr32a*^*+*^ and *PPK*^*+*^. To refine the expression of the *PPK-Gal4*, we generated a *Gr32a-LexA* that fully recapitulated the expression pattern of *Gr32a-Gal4* (Supplementary Fig. 4a). By intersecting *Gr32a* and *PPK* driver lines, we could label UNs exclusively (Fig. 4f), allowing for specific elucidation of their function. We found that silencing UNs was sufficient to block the increase of the defensive response induced by mating (Fig. 4h), while activating them promoted the defensive response in decapitated virgins (Fig. 4i). In contrast, activating UNs did not enhance the defensive response in males (Supplementary Fig. 4b), indicating that increased defensive response mediated by UNs is female-specific. Moreover, heat activation of UNs via dTRPA1 in virgin females diminished the mating success rate (Fig. 4j), suggesting that sexual receptivity is reduced in concordance with the increased defensive response after mating.

Additionally, the Ca^2+^ level in UNs’ axonal projections in the VNC in newly mated females was stronger than that in virgins and in females 8 h post-mating, arguing that UNs can be activated by mating. The effect can last for less than 8 h, consistent with behavioral results (Fig. 3d and Supplementary Fig. 4c, d).

The axons of the UNs were found to project to the abdominal ganglion (Abg) of the VNC, forming pronounced arborization along the VNC midline (Fig. 4f, g), which is distinct from that of SPSNs^34^. To further characterize these neurons, we examined the expression of *PPK*, which is known to mediate PMR. UNs expressed *PPK* (Fig. 4d), so they are different from the *PPK*^*−*^ neurons mediating copulation-induced PMR^30^. Also, the location of their cell bodies and arborization patterns in the VNC were different from those of the oviduct neurons that sense ovulation^37^. The UNs that mediate short-term PMR are thus a group of previously uncharacterized neurons.

### UNs activate LK-releasing neurons

**Fig. 5:**
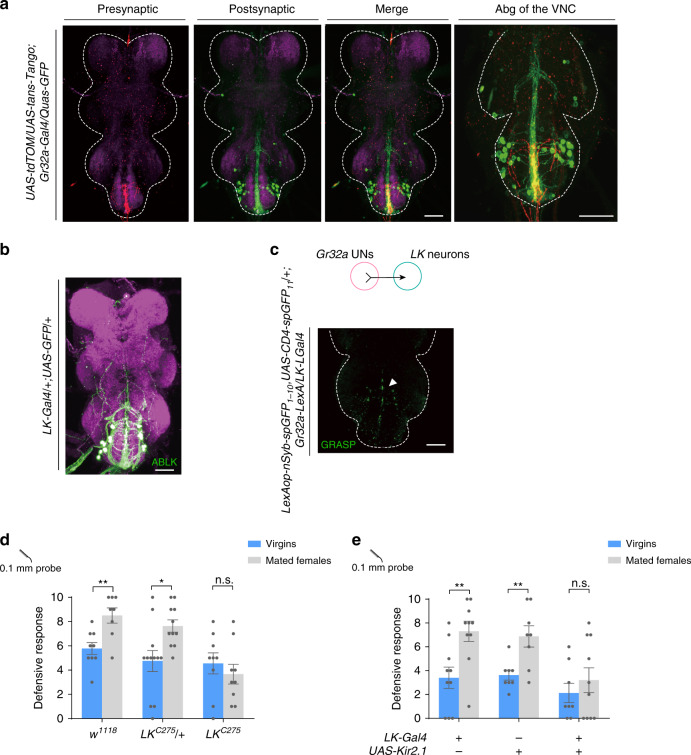
*LK* neurons function downstream of UNs to mediate increased defensive response in mated females. **a** Putative second order neurons of UNs (red) revealed by trans-Tango (green). Magenta: nc82. Right panel: the abdominal ganglion (Abg) of the VNC was zoomed in. White dashed line outlines the border of the VNC. Scale bar represented 50 μm. **b** Immunofluorescence of abdominal *LK* neurons (ABLK) (green) and nc82 (magenta). Scale bar represented 50 μm. **c** GRASP signal (green) between UNs and *LK* neurons in the Abg. White dashed line outlines the border of the VNC. Scale bar represented 50 μm. **d**, **e** Mutation of the *LK* gene **d** or silencing *LK* neurons with Kir2.1 **e** blocked the mating-induced defense facilitation. *n* = 9, 8, 12, 11, 9, 9 (from left to right) in **d**, *n* = 10, 10, 8, 8, 9, 8 (from left to right) in **e**. Error bars indicate mean ± SEM, **p < 0.01, *p < 0.05, *p* > 0.05 (n.s.), n.s., not significant, two-tailed unpaired *t* tests. Source data are provided as a Source Data file.

To map the neural circuit that relays post-mating information from UNs to higher brain centers, we used the Trans-tango method^38^, which will label the putative second order neurons of UNs (Fig. 5a). Putative postsynaptic neurons of UNs were located in the Abg of the VNC and extended ascending neurites along the VNC midline (Fig. 5a). The morphology of these neurons resembled that of the ABLK neurons in the Abg which releases leucokinin (LK) to regulate food and water intake (Fig. 5b)^39^. Indeed, intense GRASP signals were observed between UNs and ABLK neurons in the Abg (Fig. 5c). Importantly, LK was required for the post-mating modulation of the defensive response, as either mutation of the *LK* gene (Fig. 5d) or silencing of LK releasing neurons by Kir2.1 overexpression blocked mating-induced defense facilitation (Fig. 5e). These data indicated that UNs synapse with ABLK neurons to mediate the increased defense level in within 8 h post-mating females. We have also confirmed that *LK-Gal4* and *Tmc-L*^*Gal4*^ did not express in the female reproductive system (Supplementary Fig. 5b, c), suggesting that ABLK neurons played an essential role in this behavior.

### CTNs are activated by LK

**Fig. 6:**
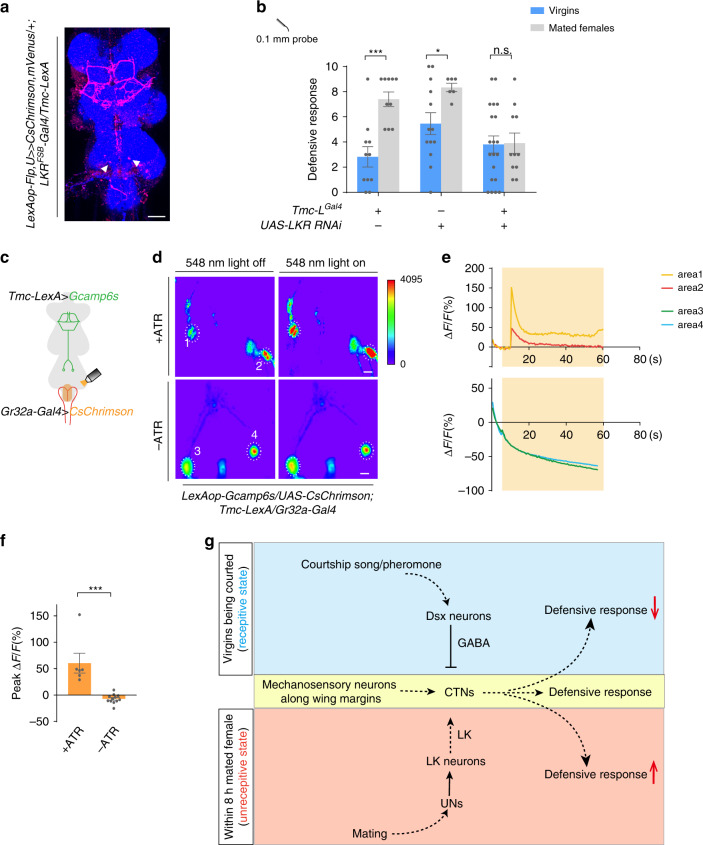
CTNs are activated by LK to induce increased defensive response in mated females. **a** Intersection between *Tmc* neurons and *LKR* neurons (red). White arrow indicated the cell bodies of CTNs. Blue: nc82. Scale bar represented 50 μm. **b** Knocking-down LKR in *Tmc-L* neurons blocked the mating-induced increase of defensive response. *n* = 11, 10, 13, 6, 20, and 11 (from left to right). ***p < 0.001, *p < 0.05, *p* > 0.05 (n.s.), n.s., two-tailed unpaired *t* tests. **c** Schematic for assays in (**d**). **d** Representative imaging of Ca^2+^ responses in CTNs’ cell bodies in the VNC before (left) and after (right) activating UNs with CsChrimson. Upper panels: flies fed with all-trans retinal (ATR). Lower panels: without ATR. The cell bodies of CTNs were cycled with white dashed lines. Numbers indicated different cell bodies. Scale bar represented 50 μm. **e** Representative traces of Δ*F*/*F* of CTNs’ cell bodies showed in (**d**) after UNs activation. **f** Ca^2+^ signal of flies supplied with ATR (left bar, *n* = 6) was elevated by UNs stimulation compared with flies without ATR (right, *n* = 12). ***p < 0.001, two-tailed Mann–Whitney nonparametric test. **g** Working model for courtship and mating mediated behavioral switch of defensive response in female flies. Error bars indicate mean ± SEM, n.s., not significant. Source data are provided as a Source Data file.

Based on the fact that activation of UNs and CTNs produced a similar increase in the defensive response (Fig. 4i and Fig. 1e), we speculated that LK released upon copulation may act directly on CTNs. To determine whether CTNs express LK receptor (LKR), we used the intersection of *Tmc-LexA* and *LKR-Gal4*. We first confirmed that *Tmc-LexA* can largely recapitulate the expression pattern of *Tmc-L*^*Gal4*^ (Supplementary Fig. 5a). One of the stained neuron groups was identified to be CTNs based on their cell body location and characteristic music stand arborizations (Fig. 6a). Furthermore, LKR expression in CTNs was functionally required for the increased post-mating defensive response as knocking-down LKR in *Tmc-L* neurons blocked the mating-induced increase in defensive response (Fig. 6b).

To provide more evidence for the potential functional connectivity between UNs and CTNs, we monitored the neuronal activity of CTNs with GCaMP6s and at the same time stimulated UNs with CsChrimson^40^ (Fig. 6c). Upon light stimulation, a dramatic Ca^2+^ elevation was detected in the cell bodies of CTNs (Fig. 6d–f), suggesting that CTNs likely receive excitatory signals from UNs. This activation was undetectable in the absence of all-*trans* retinal (ATR) that is required for CsChrimson activation in insects (Fig. 6d–f). Taken together, mating activates UNs that are a direct upstream of LK-releasing neurons in the VNC. The activation of LKR of CTNs mediates the facilitation of defense response after mating.

## Discussion

Female flies show a profound behavioral switch after mating. For example, they become hypersensitive to many sensory stimuli after mating^41–44^. In this manuscript, we have shown that the behavioral responses to tactile stimuli are also exaggerated. We found that the sensitivity to a mechanical touch on the body in female flies was tuned by different mating states. Upon touch on the wing margins, the mechanical information was relayed to CTNs to trigger the defensive response. The activity of CTNs was inhibited by a *dsx* neural circuit when male courtship cues were present; After copulation, a group of *Gr32a* neurons on the uterus were activated, culminating in the enhancement of defensive response, which presumably resulted from an elevated responsiveness of CTNs by the action of LK (Fig. 6g). The switch between pre-mating and post-mating states in the defensive response is fast and reversible and of decisive importance for female receptivity.

Previous studies demonstrate that the post-mating response (PMR) is mainly mediated by male ejaculate^17,31,34,45^. Suppression of re-mating, as a major PMR effect, emerges in two phases: the fast phase commences immediately after mating, whereas the slow phase takes hours after mating to appear and can last for as long as two weeks^17^. A recently study identified a sensory (likely mechanosensory) pathway that directly encodes mating experience to suppress re-mating immediately after mating^30^. In this study, we unraveled an additional pathway that originates from the uterus, yet is independent of SP signaling, to suppress female receptivity to mate, in which the uterine neurons (UNs) was involved in enhancing the defensive response and consequently reducing receptivity after mating. Gr32a is known to form, together with other Gr molecules, a gustatory receptor complex responsive to a wide array of bitter compounds including some male cuticular hydrocarbons that inhibits male courtship to conspecific males or females from other species^35,46^. While it’s highly plausible that UNs function as an internal sensor to detect male ejaculate, another intriguing possibility is that, during copulation, a male fly transmits to his mate certain cuticular hydrocarbons, which activate Gr32a in UNs, leading to a post-mating change in the defensive response in the recipient female.

Why do females need this alternative pathway from the uterus to the CTNs to regulate their PMR? We can envision some potential functions of this pathway: 1, As sensory neurons directly innervating the uterus, the UNs are well positioned to sense the ingredients in the seminal fluid. Besides SP, the seminal fluid contains a cocktail of proteins and other molecules^31,47^, many of which take actions on unidentified sites^48^. It’s tantalizing to hypothesize that Gr32a and UNs are involved in the detection of some of the molecules. 2, UNs may act as a compliment of the LASN pathway to mediate the short-term PMR. This notion is supported by the fact that females with their LASN silenced still had substantial experience index around 4 h after mating^30^ and ablating the *Crz* neurons can’t block the transfer of all the components of seminal fluid^49^. 3, As a downstream of UNs, ABLK neurons were found to regulate water homeostasis and food intake^50^, implicating that the activation of UNs may be linked with changes of other physiological states that can last longer that hours. Although in this study we focused on the defensive behavior, it’s conceivable that UNs’ activation may impact other post-mating behaviors such as feeding, egg-laying or aggression.

A cohort of neuropeptides mediate the PMR^17,45,51^. Leucokinin was initially found critical for body water balance as it is a neurohormone to increase Malpighian tubule fluid secretion and hindgut motility^52^. It also plays a role in the regulation of meal size^50^. Our present study may provide a link between the water/nutrition balance system and the mechanism for female receptivity via a common signaling channel mediated by LK and LKR. Although there are only a few of clusters of LK neurons in the fly^53^, LKR is expressed broadly in the fly central nervous system and other tissues^50,54,55^. Notably, among the 20 LK neurons in the VNC, at least 4–6 abdominal LK (ABLK) neurons also express DH44, a neuropeptide with established roles in female reproductive behaviors^51,52,56^. It is an open question whether there is any functional heterogeneity among the ABLK neurons. Intriguingly, the LK receptor LKR is homologous to vertebrate tachykinin receptors. In rodents, tachykinins and all neurokinin receptors are present in the uterus and their abundance is regulated during pregnancy^57^. It thus seems plausible that the LK pathway plays an evolutionarily conserved role in the reproductive behaviors of female animals.

The fly VNC has attracted less attention as a site for neural integration for controlling behavior, despite that its critical role in generating motor output is well-documented^58,59^. In this study, we unequivocally demonstrated that the mating state-dependent, and LK-mediated changes in defensive response rely on neural plasticity occurring exclusively within the VNC. The CTNs neural circuit characterized in this study provides an entry point to delineate the neural mechanism whereby ongoing behavior is fine-tuned at every moment of actions by sensory-guided neural plasticity. Interestingly, the sheet-holder shaped arborization of CTNs in the accessary mesothoracic ganglion is very similar to the structure in the same VNC region reported to be the neural substrate that balances locomotion and feeding^60^. In that study, the arborizations were visualized with *E564-Gal4*, which labels a group of brain-descending neurons involved in gustatory information processing. These descending neurons were also reported to be downstream of sensory afferents from multiple appendages. An attractive scenario is that the sheet-holder shaped arborization serves as the integrative module that coordinates feeding, locomotion and defensive response in a sexual activity-dependent manner with and/or without the brain involvement. Further study is needed to elucidate whether CTNs and *E564* neurons connect to each other, and whether the regulatory dsx and LK inputs impinge on this specific neural structure.

The neural circuits in the fly brain encoding mating rejection are poorly understood. The copulation rate or latency are commonly measured as a readout of receptivity. However, in view of the rich repertoire of the motor programs for rejection that includes kicking, fencing, wing flicking and ovipositor extrusion^9,61^, it is conceivable that a multilayered neural network distributing the brain^14^ and VNC^62,63^ operates to organize rejection behaviors^8^. One of the key neural elements controlling receptivity is the *dsx*-positive pC1 cluster in the female brain^14^, the male counterpart of which includes the P1 cluster, the neural center that makes the decision to court^28,64,65^. Although we have established a synaptic connection between a class of *dsx* neurons and CTNs, it remains to be examined whether pC1 has any role in controlling CTNs and if it does, which descending neurons deliver the pC1 output to CTNs.

## Methods

### Fly stocks

Flies were maintained at 25 °C incubator with a 12 h/12 h light cycle and humidity control unless otherwise noted. *Gad1-Gal80, sp*^*null*^*, Gr32a*^Δ^*and Gr32a*^*KO*^ were a present from Dr. Yi Rao at Peking University, China; *dsx-Gal4* and *dsx-LexA* were from Dr. Chuan Zhou at Institute of Zoology, CAS, China; *Tmc-L*^*Gal4*^ was from Dr. Yanmeng Guo (UCSF, USA), and this line was constructed by inserting Gal4 in the first exon; *PPK-LexA* was a gift from Dr. Fengwei Yu (IMCB, Singapore); *PPK-Gal4* was provided from Dr. Xin Liang at Tsinghua University, China; *LKR*^*FSB*^*-Gal4* was a gift from Dr. Justin Blau at New York University, USA. RNAi lines were all from Tsinghua Fly Center (THFC), China; The GMR lines were from the Flylight project^66^; The other lines were from Bloomington Stock Center.

### Generation of transgenic flies

*Tmc-LexA* was generated by amplifying the Tmc promoter region (3 kb) and inserting the fragment into the pBPLexAp65Uw-MCS vector^67^. Transgenic flies were made using the phiC31 site-specific integration system^68^. Primers are as follows:

Tmc-LexA forward: GCACCGGTCCTTGTTCACATCATCG

Tmc-LexA reverse: CTGGTACCGCTGCTGGTTCCTCGT

LexA forward: GCGCCTAGGATGAAGGCTCTCACGGC

LexA reverse: GCGGCATGCCAGCCAATCTCCGTTGC

*Gr32a-LexA* was generated by the HACK method^69^. pHACK-Gal4>LexA construct was generated by replacing Gal80 sequences of the pHACK-Gal4 > Gal80 with the LexA sequence. pHACK-Gal4>LexA plasmid was injected to *nos-cas9/cyo; Gr32a-Gal4/TM6B* flies using standard embryo injection procedures. Injected flies were crossed with double balancer flies, and we picked *w*^+^*RFP*^+^ flies in next generation.

### Tissue dissection, staining, and imaging

Brains and VNC of 5–7-days-old flies were dissected in dissection buffer (PBST: 0.015% triton X-100 in 1x PBS) and fixed in 4% PFA at room temperature on a shaker for 20 min, then washed for 4 × 20 min in wash buffer (0.3% triton in 1x PBS). After this, the tissues were blocked in block buffer (1x heat inactivated normal goat serum with 0.3% triton in 1x PBS) for 30 min at room temperature. The samples were then incubated with primary antibody at 4 °C overnight. The primary antibodies used were: Rabbit-GFP (Invitrogen, A11122, 1:500 dilution), Rabbit-DsRed (Rockland 39707. 1:500 dilution), Mouse-GFP (Sigma-Aldrich, G6539. 1:500 dilution), Chicken-GFP (AVES, GFP-1020. 1:500 dilution), Rabbit-GABA (Sigma-Aldrich, A2052, 1:500 dilution), Mouse-nc82 (Hybridoma Bank DSHB, Brunchpilot, 1:50 dilution). On the second day, tissues were washed for 4 × 20 min and then incubated in secondary antibodies. The secondary antibodies were all from Invitrogen and used at 1:200 dilution: Alexa Fluor 555 anti-Rabbit (A-21428), Alexa Fluor 488 anti-Mouse (A11001), Alexa Fluor 647 anti-Mouse (A-2123511031). After incubation, tissues were washed for 4 × 10 min. VNC and brains were mounted on a slide for imaging. An Olympus FV1000 microscope with 20X air lens or 40X oil-immersion lens was used for confocal imaging.

### Defensive response assay of virgins

Flies of 7–10-day old were used in behavioral experiments. Flies were anesthetized on ice and recovered for 30 min before test. Behaviors were video-recorded with a high-speed camera (Basler ORBIS OY-A622f-DC) for offline analysis. All behavioral experiments were done at 25 °C and 60% humidity except for the heat activation experiments.

For experiments on intact flies, flies were kept ventral-side down on ice and their notum was attached to an insect pin by low-melting-point wax. The head and thorax were fixed together to reduce movement. The pin was inserted into a piece to clay. For decapitated flies, their heads were cut with spring scissors (Fine Science Tools) on ice. Headless flies were transferred into a 10 mm × 35 mm culture dish (CORNING) with wet filter paper on bottom^12^.

We used plastic fibers as probe to stimulate flies’ wings between wing coastal and L2 vein from proximal to distal to mimic the touch by male. The interval of two stimulus was 1 s, and the stimulation was given to left wing or right wing alternatively. Kicking times out of ten trials were counted as defensive response.

### cVA painting behavior

0.2 μg cVA (Sigma) dissolved in 10% ethanol was applied to filter paper and evaporated. Eight to sixteen intact wild-type (*w*^*1118*^) virgins were gently vortexed with the filter paper twice for 20 s, roamed for 30 min prior to defense assays (as described above)^70^. Control virgins were vortexed with filter paper applied with 10% ethanol only.

### Defensive response assay of mated females

Virgins were kept in groups for 7–10 days at 25 °C, all males were raised individually. Virgins of identical genotype were aspirated in to courtship chamber with 4 mm height and 10 mm diameter. Half of the virgins were paired with 5–7-day male *Canton S* for 30 min. After mating, virgins and mated females were taken out simultaneously, and we compared the defensive response (as described above) of virgins and mated females under the same preparation. In Fig. 3d, matched females represented that virgins were placed solely in courtship chambers paralleled with mated females under same preparation. Before doing defense assay, matched females and already mated females were kept on food for certain hours.

In Fig. 4b, 7–10-day virgins were paired with naive male *Canton S* in 2 mm × 10 mm × 10 mm courtship chamber, and courtship was record. We analyzed male touching times and virgin defensive response. Then, we suctioned mated male and paired new naïve *Canton S* to mated female. Courtship to mated female was record for 15 min, and male touching times and mated female defensive response were analyzed. Defensive response ratio was calculated as mated female or virgin kicking responses/naïve male touching times.

### Optogenetics and thermogenetics

Newly eclosed flies were collected and transferred into food containing a piece of filter paper with 500 µl of sugar-retinal solution (500 µM all-trans-retinal diluted in 100 mM sucrose solution) on surface^71^. Flies were kept in dark and used for optogenetics experiments after fed with all-trans-retinal 3 to 5 days.

For heat activation experiments, flies were raised in an incubator of 22 °C. All experiments were done on a metal bath at 30 °C.

### Female receptivity assay

Flies of both sexes were collected after eclosion. 7–10-day virgin females were kept in groups and 5–7-day males were kept individually. Virgins and naïve males were paired in courtship chamber (described as above). Each pair was recorded for 30 min. Time to copulation (time before courtship start) and successful rates were analysed.

### Simulative courtship assay

Five *Canton. S* males were placed into a 1-cm wide chamber with a copper mesh cover. The female flies attached to an insect pin were placed 10 cm above a speaker (JMC) and 1 cm in front of the chamber with males. Particle velocity of the courtship song varied between about 0.87 mm/s and 4.90 mm/s. We measured sound levels with the sound-level meter positioned at the center of our speaker, with a distance of 3 cm to the speaker^72^. The defensive response was tested in this environment. The courtship song was download from: https://www.youtube.com/watch?v=KzWIuhXMUko. For assay with male flies, a male was attached to an insect pin and placed 1 cm away in front of a glass tube (Φ5 mm × 10 mm) containing a free-moving virgin female.

### Calcium imaging

The entire VNC was dissected out in a recording chamber containing external solution (103 mM NaCl, 3 mM KCl, 5 mM TES, 10 mM trehalose, 10 mM glucose, 26 mM NaHCO_3_, 1 mM NaH_2_PO4, and 4 mM MgCl_2_, pH 7.25, 310 mOsm). 2 mM CaCl_2_ was added to the saline before use.

Calcium imaging was performed using an Olympus BX51WI microscope with a 40X water immersion objective, an Andor Zyla camera and a Uniblitz shutter. The Ca^2+^ indicator GCaMP6 was used to measure the Ca^2+^ signal. GCaMP6 was excited with 488 nm light and the fluorescent signals were collected at 5 Hz. ATP solution was added into the chamber to a final ATP concentration of 10 mM^73^. Ca^2+^ signal was collected before, during and after the application of ATP. ROIs were manually selected from the cell body area with ImageJ. Fluorescent change was calculated as %Peak Δ*F*/*F*_0_ = (*F*_peak_ − *F*_0_)/*F*_0_ × 100, where *F*_0_ corresponds to the average intensity of 20 frames of background-subtracted baseline fluorescence before ATP application and *F*_peak_ corresponds to the highest fluorescence after ATP application.

For optigenetic activation, VNC was prepared as described above. Images were acquired on an Olympus FV1000 confocal microscope using a 40 X Water lens. GCaMP6 was excited with 488 nm light and CsChrimson was activated with 546 nm light. Green fluorescent signals were collected at 5 Hz and 546 nm light was applied after capturing 20 frames. All operation was automatically controlled by the FV10-ASW 3.0 software. ROIs were manually selected from the cell body area with ImageJ. Fluorescent change was calculated as %Δ*F*/*F*_0_ = (*F*_t_-*F*_0_)/*F*_0_ × 100, where *F*_t_ corresponds to resulting fluorescence value for each time point and *F*_0_ corresponds to average of 20 frames of background-subtracted baseline fluorescence before 546 nm stimulation.

For imaging of post-mating activity of Gr32a neurons, each virgin female was pair with a C.S. male in a courtship chamber until they finished copulation. The female flies were either imaged immediately or transferred to a food vial for later imaging.

To image the neuronal terminals at the VNC, the female fly was immobilized ventral side down with two insect pins on a Sylgard-coated dish. The legs were removed and a small window was cut between the hypopleura. The fat body and sacs were gently removed to expose the VNC. The preparation was then placed underneath an Olympus BX51WI microscope with a 40X water immersion objective. All images were taken with the same settings.

### Statistics and reproducibility

Experimental flies and genetic controls were tested at the same condition, and data are collected from at least two independent experiments. Statistical analysis was performed with GraphPad Prism Software version 6.01 (GraphPad Software). All data in bar and line graphs are expressed as means ± SEMs. Two-tailed unpaired *t* tests and two-tailed Mann–Whitney nonparametric test were used to evaluate the statistical significance between two datasets. Mating rates were analyzed with Fisher’s exact test.

### Reporting summary

Further information on research design is available in the Nature Research Reporting Summary linked to this article.

## Supplementary information

Supplementary Information

Reporting Summary

## Data Availability

We declare that all data supporting the findings of this study are available within the article and its Supplementary Information files or from the corresponding author upon reasonable request. Source data are provided with this paper.

## Source data

Source Data
