# Supplementary material for: A neural circuit encoding mating states tunes defensive behavior in Drosophila
Source: Nat Commun. 2020 Aug 7;11:3962. doi: 10.1038/s41467-020-17771-8 (PMC7414864; doi:10.1038/s41467-020-17771-8)
Supplement: Supplementary file 1 — Supplementary Information [file 41467_2020_17771_MOESM1_ESM.pdf]

A Neural Circuit Encoding Mating States Tunes Defensive Behavior in *Drosophila*

Liu et al.

Supplementary Information

Supplementary Figures and Legends

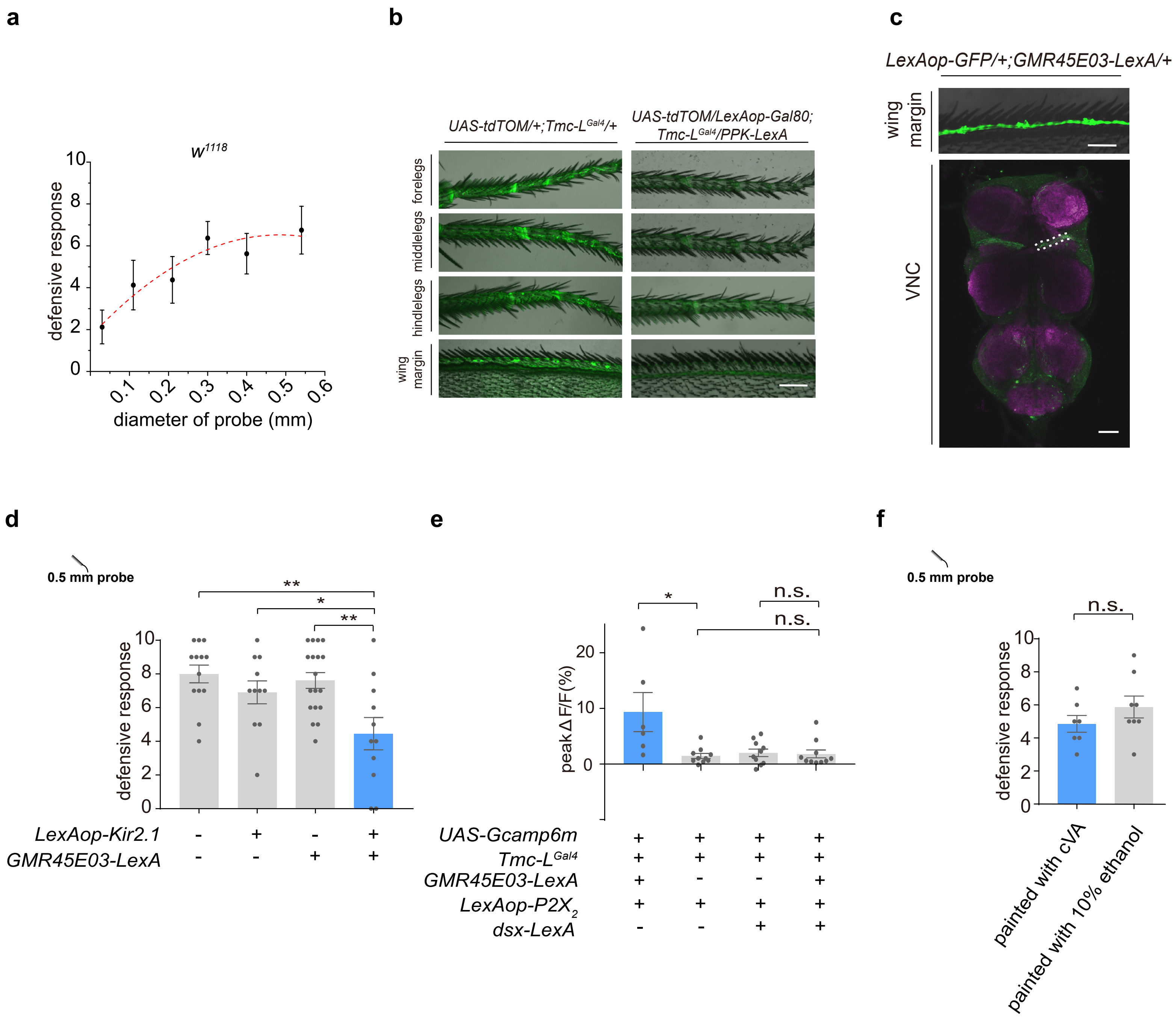

Supplementary Fig. 1 Supplied experiments to Fig.1. **(a)** Defensive response increased with the diameter of the probe. *n*= 8 for each group. **(b)** Peripheral expression of Tmc-L (left). Peripheral expression of Tmc-L was eliminated by *PPK-lexA>LexAop-Gal80* (right). Scale bar represented 50 μm. **(c)** Immunofluorescence of *GMR45E03-LexA* (green) and NC82 (magenta) in the wing margin (above) and VNC (below). This driver line labeled mechanoreceptors on the wing margin which projected to accessary mesothoracic ganglion (highlighted with white dashed line). Scale bar represented 50 μm. **(d)** Blocking *GMR45E03-LexA* neurons' activity with Kir2.1 largely reduced the defensive response against wing margin touch. *n*= 13, 11, 18 and 11 for each group. $^{**}p < 0.01$, $^{*}p < 0.05$, two-tailed unpaired *t* tests. **(e)** Calcium response of CTNs' cell bodies in additional control groups. *n*= 5, 10, 10 and 10 for each group. $^{*}p < 0.05$, $p > 0.05$ (n.s.), two-tailed Mann–Whitney nonparametric test. **(f)** Defensive response score of virgins painted with cVA and 10% ethanol stimulated by 0.5mm probe. *n*= 7 and 8 for each group. $p > 0.05$ (n.s.), two-tailed unpaired *t* tests.Error bars indicate mean ± SEM, n.s., not significant. Source data are provided as a Source Data file.

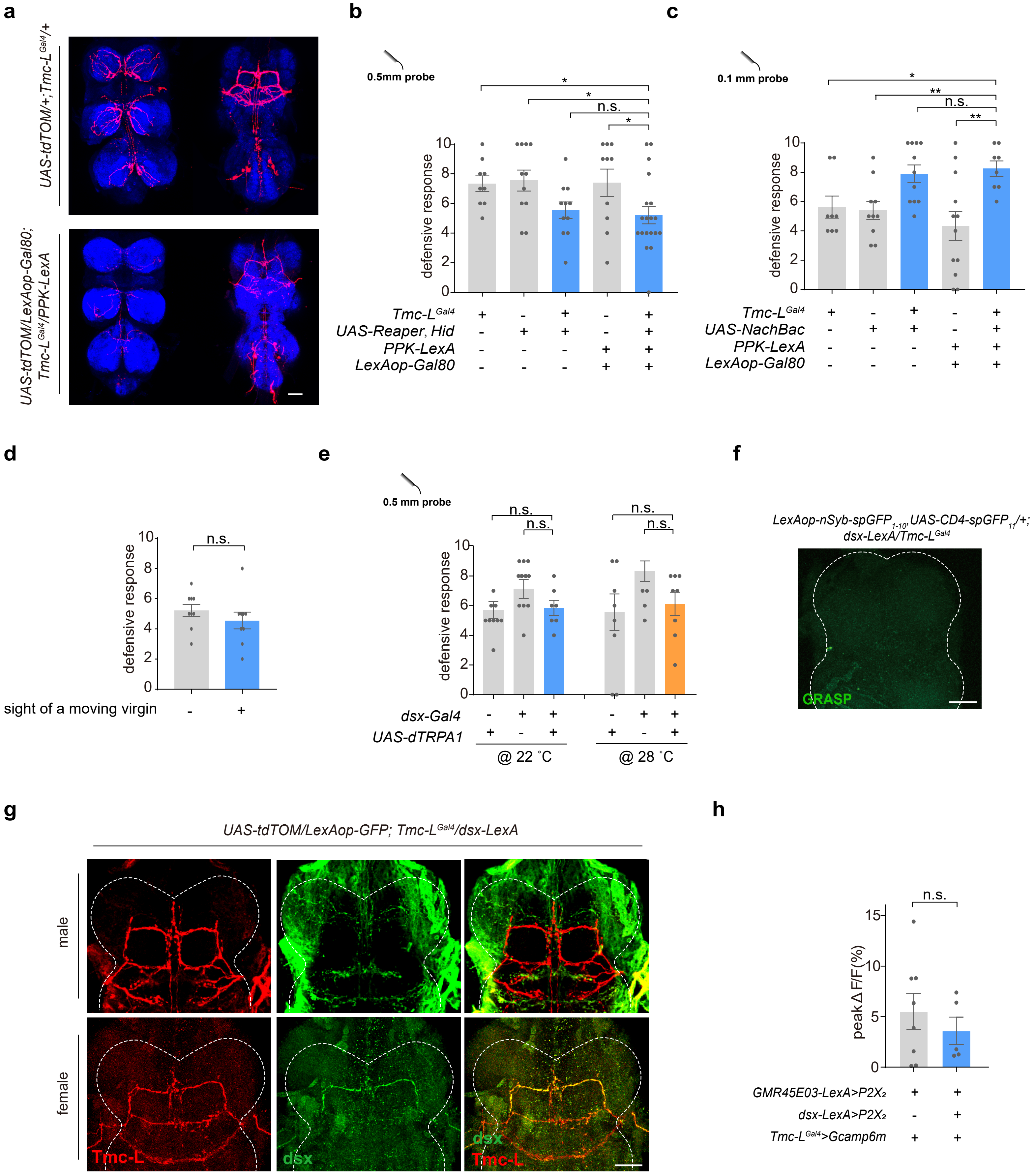
a
UAS-tdTOM/+;Tmc-L^Gal4/+
UAS-tdTOM/LexAop-Gal80; Tmc-L^Gal4/PPK-LexA
b
0.5mm probe
defensive response
Tmc-L^Gal4
UAS-Reaper, Hid
PPK-LexA
LexAop-Gal80
c
0.1 mm probe
defensive response
Tmc-L^Gal4
UAS-NachBac
PPK-LexA
LexAop-Gal80
d
defensive response
sight of a moving virgin
e
0.5 mm probe
defensive response
dsx-Gal4
UAS-dTRPA1
@ 22 °C
@ 28 °C
f
LexAop-nSyb-spGFP1-10, UAS-CD4-spGFP11/+; dsx-LexA/Tmc-L^Gal4
GRASP
g
UAS-tdTOM/LexAop-GFP; Tmc-L^Gal4/dsx-LexA
male
female
Tmc-L
dsx
h
peak ΔF/F(%)
GMR45E03-LexA>P2X2
dsx-LexA>P2X2
Tmc-L^Gal4>Gcamp6m

Supplementary Fig. 2 CTNs' role in male flies. **(a)** Immunofluorescence of Tmc-L (red) and NC82 (blue) in the ventral and dorsal VNC in males. Below: the expression pattern of CTNs, and the peripheral projections of Tmc-L in the ventral VNC were eliminated by *PPK-Gal80*. Scale bar represented 50 μm.**(b,c)** CTNs of males coordinated defensive response. Ablating all Tmc-L neuron or only CTNs with Reaper and Hid (b), and activating all Tmc-L neuron or only CTNs with NachBac (c). *n*= 9, 11, 11, 10 and 19 for each group in (b), *n*= 8, 10, 11, 12 and 8 for each group in (c). $^{**}p < 0.\ 01$, $^{*}p < 0.\ 05$, $p > 0.05$ (n.s.), two-tailed unpaired *t* test. **(d)** Defensive response of males with the sight of moving virgins. *n*= 9 for each group. $p > 0.05$ (n.s.), n.s., two-tailed unpaired *t* test. **(e)** Activating *dsx* neuron with dTrpA1 didn't induce significant difference of defensive response against mechanical stimuli in males. *n*= 10, 14, 7, 9, 9 and 8 (from left to right). $p > 0.05$ (n.s.), two-tailed unpaired *t* test. **(f)** No GRASP signal was observed between dsx neurons and CTNs in males. White dashed line outlines the border of the mesothoracic ganglion of the VNC. Scale bar represented 50 μm. **(g)** Co-expression of *dsx* neurons and *Tmc-L* neurons in the dorsal metathoracic ganglion of the VNC in male (upper) and female (lower). White dashed line outlines the border of the mesothoracic ganglion of the VNC. Scale bar represented 50 μm. **(h)** Peak fluorescence changes (ΔF/F0) of CTNs' cell bodies in the VNC after activating wing margin mechanosensory neurons via $P2X_2$ in males. Activating wing margin mechanosensory neurons elicited elevated calcium signal of CTNs (left bar, *n*= 8). Activating wing margin mechanosensory neurons and dsx neurons simultaneously didn't decrease calcium signal of CTNs (right bar, *n*= 5). $p > 0.05$ (n.s.), two-tailed unpaired *t* test. Error bars indicate mean ± SEM, n.s., not significant. Source data are provided as a Source Data file.

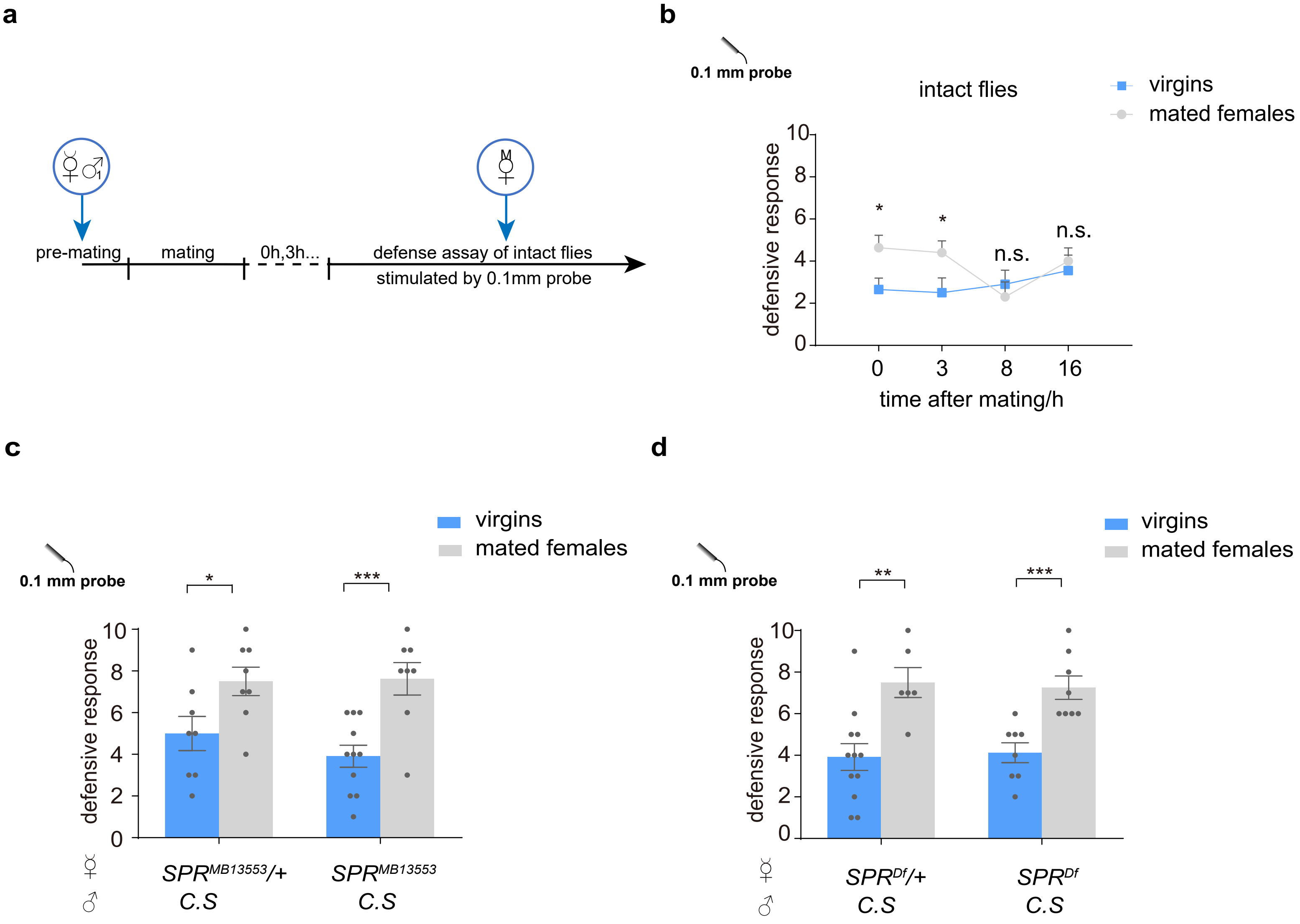

Supplementary Fig. 3 **I**ncreased defensive response induced by mating can last for hours in intact flies, which was independent with SP. **(a)** Schematic for assays in (b). Males were removed after copulation, and mated females were kept for certain hours before the defensive response assay. **(b)** The intact mated female flies exhibited a higher defensive response to mechanical stimuli on the wing margins compared to virgin females, consistent with decapitated flies (see in Fig. 3d). Matched females represented that virgins were placed solely in courtship chambers paralleled with mated females under the same preparation. *n*= 20, 10, 10 and 9 for each group of matched virgins, *n*= 22, 10, 10 and 8 for each group of mated virgins. **p* < 0.05, *p* > 0.05 (n.s.), two-tailed unpaired *t* test. **(c, d)** Female flies of *SPR* mutant alleles, $SPR^{MB13553}$ (c) and SPR^{Df} (d), showed an increase of defensive response after mating. *n*= 8, 8, 11 and 8 for each group in (c), *n*= 12, 6, 8 and 8 for each group in (d). ****p* < 0.001, ***p* < 0.01, **p* < 0.05, two-tailed unpaired *t* test. Error bars indicate mean ± SEM, n.s., not significant. Source data are provided as a Source Data file.

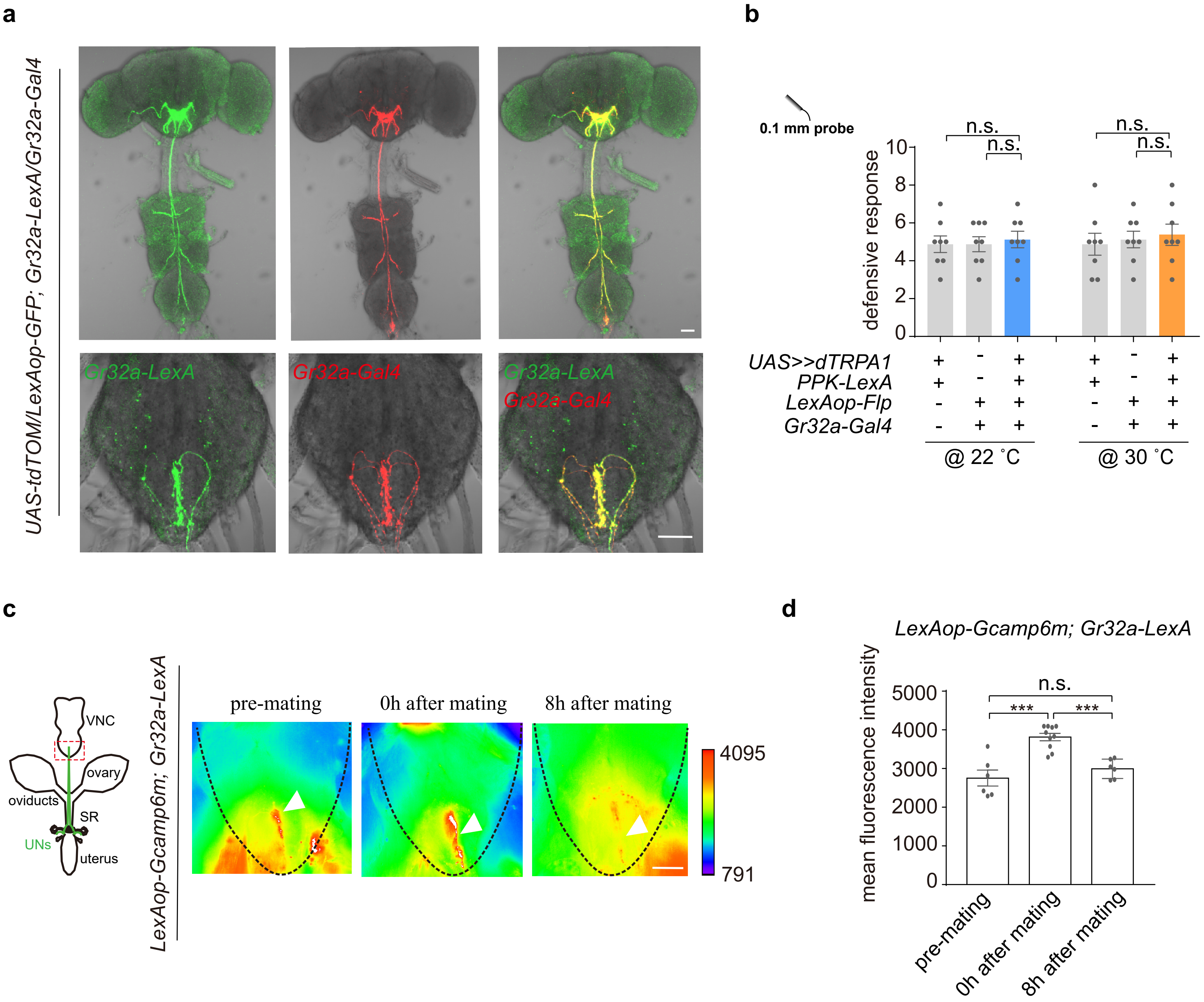

Supplementary Fig. 4 UNs could be activated by mating to modulate defensive response, which was female-specific. **(a)** Co-expression of *Gr32a-Gal4* and *Gr32a-LexA* in the CNS (upper) and in the Abg of the VNC (lower). Scale bar represented 50 μm (upper) and 25 μm (lower). (b) Activating UNs in decapitated males at 22°C and 30°C. *n*= 8 for each group. $p > 0.05$ (n.s.), two-tailed unpaired *t* test. (c) Representative imaging of Ca^{2+} responses in neuronal projections of UNs in the Abg from virgins (left), newly mated (middle) and females 8h post-mating (right). Left panel: schematic showed that the region was imaged in right panels. Scale bar represented 25 μm. (d) Mean fluorescence intensity of Ca^{2+} signal in neuronal projections of UNs in the Abg from virgins, newly mated and 8h post-mating females. *n*= 6, 11 and 6 for each group. ***$p < 0.001$, $p > 0.05$ (n.s.), two-tailed unpaired *t* test. Error bars indicate mean ± SEM, n.s., not significant. Source data are provided as a Source Data file.

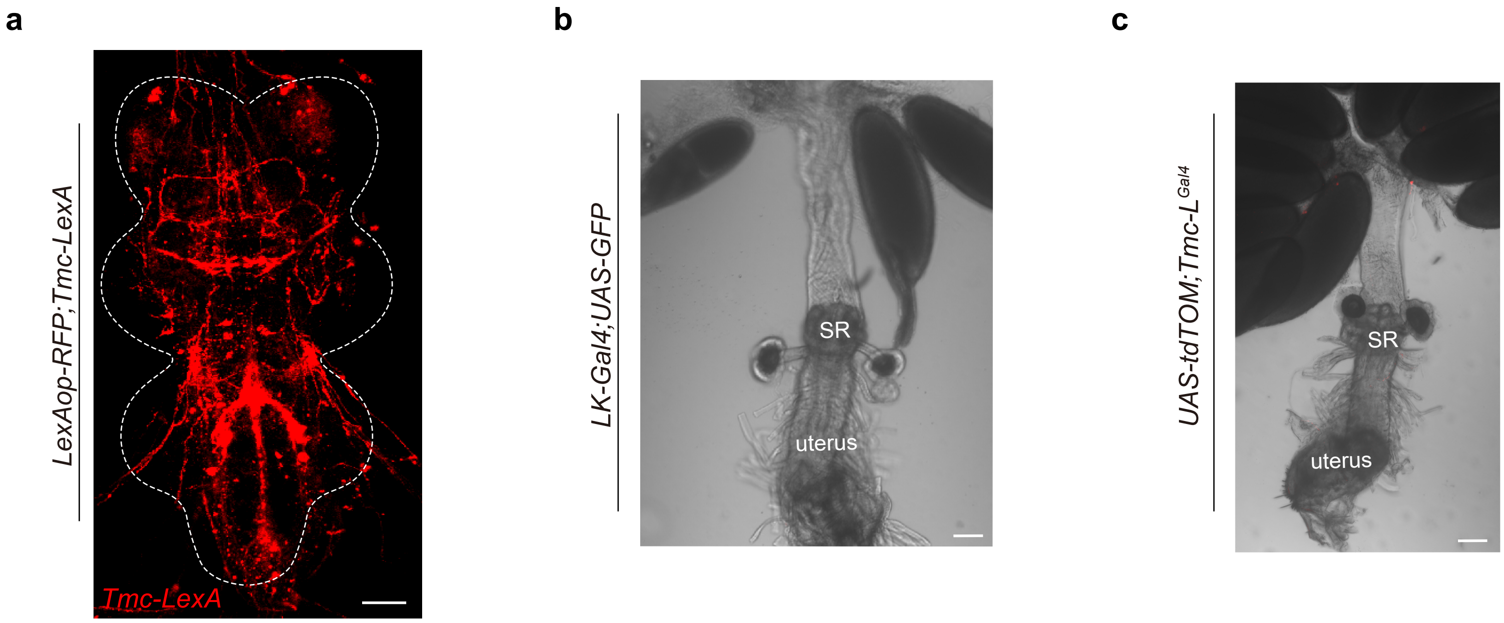

Supplementary Fig. 5 The expression patterns of LK-Gal4 and Tmc-L^{Gal4} in female reproductive system. **(a)** Expression patterns of *Tmc-LexA* in the VNC. Scale bar represented 50 μm. **(b-c)** Expression patterns of *LK-Gal4* (b) and *Tmc-L^{Gal4}*. (c) in female reproductive system. Scale bar represented 50 μm.
